# C4b-binding protein α-chain enhances antitumor immunity by facilitating the accumulation of tumor-infiltrating lymphocytes in the tumor microenvironment in pancreatic cancer

**DOI:** 10.1186/s13046-021-02019-0

**Published:** 2021-06-24

**Authors:** Kosuke Sasaki, Shigetsugu Takano, Satoshi Tomizawa, Yoji Miyahara, Katsunori Furukawa, Tsukasa Takayashiki, Satoshi Kuboki, Mamoru Takada, Masayuki Ohtsuka

**Affiliations:** Department of General Surgery, Chiba University, Graduate School of Medicine, 1-8-1, Inohana, Chuo-ku, Chiba City, Chiba, 260-8677 Japan

**Keywords:** C4BPA, pancreatic cancer, CD40, CD8, T cell antitumor immunogenicity

## Abstract

**Background:**

Recent studies indicate that complement plays pivotal roles in promoting or suppressing cancer progression. We have previously identified C4b-binding protein α-chain (C4BPA) as a serum biomarker for the early detection of pancreatic ductal adenocarcinoma (PDAC). However, its mechanism of action remains unclear. Here, we elucidated the functional roles of C4BPA in PDAC cells and the tumor microenvironment.

**Methods:**

We assessed stromal C4BPA, the C4BPA binding partner CD40, and the number of CD8^+^ tumor-infiltrating lymphocytes in resected human PDAC tissues via immunohistochemical staining. The biological functions of C4BPA were investigated in peripheral blood mononuclear cells (PBMCs) and human PDAC cell lines. Mouse C4BPA (mC4BPA) peptide, which is composed of 30 amino acids from the C-terminus and binds to CD40, was designed for further *in vitro* and *in vivo* experiments. In a preclinical experiment, we assessed the efficacy of gemcitabine plus nab-paclitaxel (GnP), dual immune checkpoint blockades (ICBs), and mC4BPA peptide in a mouse orthotopic transplantation model.

**Results:**

Immunohistochemical analysis revealed that high stromal C4BPA and CD40 was associated with favorable PDAC prognosis (*P*=0.0005). Stromal C4BPA strongly correlated with the number of CD8^+^ tumor-infiltrating lymphocytes (*P*=0.001). In *in vitro* experiments, flow cytometry revealed that recombinant human C4BPA (rhC4BPA) stimulation increased CD4^+^ and CD8^+^ T cell numbers in PBMCs. rhC4BPA also promoted the proliferation of CD40-expressing PDAC cells. By contrast, combined treatment with gemcitabine and rhC4BPA increased PDAC cell apoptosis rate. mC4BPA peptide increased the number of murine T lymphocytes *in vitro* and the number of CD8^+^ tumor-infiltrating lymphocytes surrounding PDAC tumors *in vivo*. In a preclinical study, GnP/ICBs/mC4BPA peptide treatment, but not GnP treatment, led to the accumulation of a greater number of CD8^+^ T cells in the periphery of PDAC tumors and to greater tumor regression than did control treatment.

**Conclusions:**

These findings demonstrate that the combination of GnP therapy with C4BPA inhibits PDAC progression by promoting antitumor T cell accumulation in the tumor microenvironment.

**Supplementary Information:**

The online version contains supplementary material available at 10.1186/s13046-021-02019-0.

## Background

Pancreatic ductal adenocarcinoma (PDAC) is a highly malignant tumor and is unusually resistant to chemotherapy, radiotherapy, and immunotherapy [1, 2]. PDAC exhibits the tumor microenvironment (TME) characterized by an extensive desmoplastic stroma and a cellular infiltrate composed of immunosuppressive leukocyte populations [3, 4]. We have identified a component of the complement, C4b-binding protein α-chain (C4BPA), as a novel serum biomarker for detecting PDAC at an early stage using a comprehensive proteomic approach [5]. However, its mechanism of action remains unclear.

C4BPA is a regulatory component of the classical pathway that is synthesized by liver cells and activated monocytes [6–8]. CD40 belongs to the tumor necrosis factor superfamily of receptors and is mainly expressed on antigen-presenting cells (APCs) like B cells, dendritic cells, and macrophages [9]. In malignant tumors, CD40 expression is ubiquitously observed in most B cell malignancies and various solid tumors [10]. CD40 ligand (CD40L) is a binding partner of CD40 and is predominantly expressed on activated CD4^+^ T cells [11]. The CD40–CD40L interaction between B cells and activated T cells is essential for responses to T cell-dependent antigens, including antitumor T cell activation [12–15].

As most PDACs exhibit low T cell infiltration and are designated as immunologically “cold”, single-agent immune checkpoint blockade (ICB) therapy is not effective in PDAC patients [16]. To increase responsiveness to checkpoint blockade, there is a need to convert immunologically “cold” to “hot” tumors that have robust T cell infiltration [17]. Agonistic CD40 monoclonal antibodies (αCD40) mimic CD40L *in vivo* and have been shown to enhance the immunogenicity of cancer vaccines and trigger the regression of highly immunogenic tumors [15, 18–20]. αCD40 can reportedly drive T cell responses, reduce tumor rejection in chemotherapy [21–27], and synergize with ICB [28, 29]. C4BPA directly binds to and activates CD40, and they do not compete for B cell binding because C4BPA and CD40L bind on distinct CD40 sites. Thus, C4BPA mimics CD40L in causing B cell activation [30].

Herein, we hypothesized that C4BPA exhibits an antitumor T cell response with the accumulation of tumor-infiltrating lymphocytes (TILs) via the C4BPA-CD40 axis in PDAC. In this study, we highlighted the role of C4BPA in the acceleration of T cell antitumor immunogenicity in the TME of PDAC. These data provide novel insight into the immunologic antitumor response in the TME of PDAC and a new platform for multidisciplinary therapeutics.

## Methods

### Human tissue samples

PDAC tissues were obtained from 171 consecutive patients who underwent pancreatectomy at the Department of General Surgery, Chiba University Hospital, Japan from January 2010 to December 2014. All patients were histologically diagnosed with primary invasive PDAC. Whole tissue lysates were extracted from 5 pairs of PDAC and adjacent normal pancreas tissues resected in 2019. The study protocol was approved by the Ethics Committees of Chiba University (protocol #2958) and written informed consent was obtained from each patient before operation.

### Human and murine cell lines and culture conditions

The human PDAC cell lines BxPC-3, MIA PaCa-2, PANC-1, Capan-2, AsPC-1, Hs766T, CFPAC-1, and Capan-1 were obtained from the American Type Culture Collection (Manassas, VA, USA). The BxPC-3, MIA PaCa-2, PANC-1, and Hs766T cell lines and all the mouse pancreatic cell lines were cultured in Dulbecco’s modified Eagle medium (DMEM; Sigma-Aldrich, St. Louis, MO, USA) with 10% fetal bovine serum (FBS) and antibiotics (1% penicillin and streptomycin). Capan-2 cells were cultured in McCoy’s 5A Medium (Cytiva, Issaquah, WA, USA) with 10% FBS and antibiotics. AsPC-1 cells were cultured in RPMI-1640 medium (Thermo Fisher Scientific, Waltham, MA, USA) with 10% FBS and antibiotics. CFPAC-1 cells were cultured in Iscove’s modified Dulbecco’s medium (Thermo Fisher Scientific) with 10% FBS and antibiotics. Capan-1 cells were cultured in DMEM with 20% FBS and antibiotics.

Murine PanIN cells (KC), PDAC cells (KPC: KPC1 and 2), and paired metastasis (KPCLiv: KPC1Liv and KPC2Liv) cell lines were provided by Dr. Sunil Hingorani (University of Washington). In brief, a KC cell line was established from primary PanIN cells from a genetically engineered mouse model (GEMM) of PanIN (KC mouse: *Pdx1-cre;LSL-Kras*^*G12D/+*^) and KPC cell lines were established from primary PDAC of a GEMM of PDAC (KPC mouse: *Pdx1-cre;LSL-Kras*^*G12D/+*^*;p53*^*R172H/+*^). Further, the KPCLiv cell lines were isolated from paired liver metastases arising in KPC mice. PKCY (PKCY1 and 2) cells, derived from *Pdx1-cre;LSL-Kras*^*G12D/+*^*;p53*^*fl/+*^*;R26*^*YFP*^ mice (PKCY mice), were provided by Dr. Andrew D. Rhim (The University of Texas MD Anderson Cancer Center).

### Immunohistochemical and immunofluorescence staining

Immunostaining was performed following standard protocols. Briefly, paraffin-embedded tissue blocks were cut into sections with a thickness of 4 μm. Antigen-retrieved and protein-blocked slides were incubated with specific antibodies overnight at 4 °C. Envision^TM+^ Kits (Agilent, Santa Clara, CA, USA), VECTASTAIN® Elite® ABC Kit (Vector Laboratories, Inc., Burlingame, CA, USA) or appropriate horseradish peroxidase (HRP)-conjugated secondary antibodies were used for detecting the primary antibodies. The slides were then developed with 0.01% 3,3-diaminobenzidine. Slides were counterstained with hematoxylin and eosin (H&E) and dehydrated in ethanol and xylene. The staining patterns of C4BPA and CD40 in resected human PDAC tissues were scored as follows: low expression, staining intensity of stroma around cancer cells less than the that of islet cells (internal positive control); and high expression, staining intensity of stroma around cancer cells greater than or equal to that of islet cells. The staining patterns of CD8 in resected human PDAC tissues were scored by counting tumor stromal lymphocytes with positive staining in an average of 5 different high-power fields (HPFs) (×400). The staining patterns were classified as above and below the median (range: 2–48, median: 16, average: 17.1). The immunohistochemical staining score was evaluated independently by three investigators. The primary and secondary antibodies are listed in Supplementary Table 3.

### RNAi transfection

CD40 siRNAs (CD40 siRNA1: Cat. #SI00024213, CD40 siRNA2: Cat. #SI00024227) and control siRNA (AllStars negative control siRNA) were purchased from Qiagen (Hilden, Germany). Cells (4.0 × 10^5^) were plated in a 60-mm dish and incubated for 24 h. Cells were then treated with siRNA (5 nM final concentration) in Lipofectamine^TM^ RNAiMAX Transfection Reagent (Invitrogen, Carlsbad, CA, USA). Cells were detached and replated for the cell proliferation assay after 24 h of siRNA treatment. Protein was harvested from cells after culturing for 72, 96, 120, and 144 h.

### Cell proliferation assay

Human or mouse primary pancreatic cell lines were cultured in 96-well plates and incubated in 5% CO_2_ at 37 °C. Cell Counting Kit-8 (CCK-8; Dojindo Laboratories, Kumamoto, Japan) was used to assess cell proliferation following the manufacturer’s protocol. The absorbance was measured on a microplate reader at a wavelength of 450 nm. Each experiment was performed independently in quadruplicate for a total of three experiments. *P* < 0.05 was considered statistically significant (Mann–Whitney–Wilcoxon test) and the error bars represent the standard error of the mean (SEM).

### Western blot analysis

Total protein was purified from cultured cells using radioimmunoprecipitation assay (RIPA) buffer (Sigma-Aldrich) and preserved at -80 °C. Twenty micrograms of protein was loaded onto a 5–12.5% XV PANTERA Gel (DRC, Tama, Tokyo, Japan) and transferred onto a polyvinylidene difluoride membrane. The membranes were blocked at room temperature for 60 min in 5% milk in 0.1% Tris-buffered saline with Tween-20 (TBS-T). Membranes were incubated with anti-C4BPA (Abcam, *Cambridge*, United Kingdom), anti-CD40 (GeneTex, Irvine, CA, USA), and anti-β-actin (Cell Signaling Technology, Danvers, MA, USA) antibodies overnight at 4 °C, washed three times for 5 min each with TBS-T, and incubated with secondary antibodies. The membranes were developed using ImageQuant LAS-4000UV mini Mac (Cytiva) after immersion in the detection reagent. Western blots were quantified by densitometry analysis and normalized to β-actin using ImageJ software. A list of antibodies is shown in the Supplementary Information.

### Isolation of human and murine PBMCs and culture conditions

Peripheral blood from healthy volunteers was obtained by phlebotomy in heparinized tubes. PBMCs were isolated by centrifugation on Ficoll-Paque PLUS (Cytiva), according to the manufacturer’s instructions. Cells were washed with phosphate-buffered saline (PBS) and plated in RPMI-1640 medium (Thermo Fisher Scientific) with 10% FBS and antibiotics. PBMC suspensions were aliquoted into plastic tissue culture plates and incubated for 2 h at 37 °C in a 5% CO_2_ incubator. After transfecting nonadherent cells and washing with PBS, the nonadherent cells (lymphocytes) were cultured for 3 days in RPMI-1640 medium (Thermo Fisher Scientific) with 10% FBS and antibiotics. The cells were supplemented with 0.5% concanavalin A as a T cell mitogen and stimulated with or without recombinant human C4BPA (rhC4BPA) (Abnova, Taipei, Taiwan, 50 ng/mL) or human CD40L (Peprotech, Rocky Hill, NJ, USA, 500 ng/mL). Differentiated CD4^+^ or CD8^+^ T cells were washed and analyzed by flow cytometry. For murine PBMCs (mPBMCs), murine peripheral blood was obtained from 12 mice by cardiac puncture. mPBMCs were isolated and cultured in the same manner as human PBMCs (hPBMCs). The culture medium was RPMI-1640 medium with 10% FBS and antibiotics, without concanavalin A, and supplemented with or without mouse C4BPA peptide.

### Flow cytometry analysis

One million cells were suspended in 100 μL PBS and incubated with antibodies for 60 min on ice in the dark. The antibodies used were PerCP anti-human CD3, FITC anti-human CD4, PE anti-human CD8, PE anti-mouse CD4, and FITC anti-mouse CD8a (BioLegend, *San Diego*, CA, USA). After washing with PBS, the cells were resuspended in 500 μL PBS and measured using a CANTO II system (Beckton-Dickinson, Franklin Lakes, NJ, USA). All data were analyzed using FlowJo v10.7.1 software (Ashland, OR, USA).

### Cytotoxicity assay

PANC-1 and Capan-2 cells were analyzed for gemcitabine (Gem) exposure with or without rhC4BPA by Cell Counting Kit-8 and an annexin V FITC/propidium iodide double-staining method. After 24 h of siRNA treatment, the PDAC cells were exposed to rhC4BPA for 72 h and Gem for 24 h. The cells were then used to assess cell proliferation by CCK-8. Furthermore, after exposure to rhC4BPA for 54 h and Gem for 6 h, the PDAC cells were collected and subjected to flow cytometry. By using an Annexin V-FITC Apoptosis Detection Kit (Nacalai Tesque, Kyoto, Japan), the apoptotic cells were identified by flow cytometry. A minimum of 10,000 cells were analyzed. Cells were detached and replated for the cell proliferation assay after 24 h of siRNA treatment.

### Orthotopic transplantation model

In the orthotopic transplantation model, 8–10-week-old female C57BL/6JJcl mice (CLEA Japan, Tokyo, Japan) were injected with 2 × 10^5^ PKCY cells suspended in 25 μL DMEM/10% FBS into the subcapsular space of the pancreatic tail under anesthesia. The study protocols were described previously. Mice were euthanized on a predetermined day and the primary pancreatic tumor and the whole liver were removed. To define the baseline tumor volume, the mice were sacrificed 11 days after cell injection and the mean tumor volumes were measured. For histological analysis, the resected pancreas and livers were fixed in 4% paraformaldehyde for 24 h, transferred to 100% ethanol, and embedded in paraffin. Some parts of the specimens were embedded in tissue freezing medium (Tissue Tek O.C.T. compound, Sakura Finetek, Tokyo, Japan), placed in liquid nitrogen, stored at -80 °C, and sectioned (5 μm) using a cryostat (Leica, Wetzlar, Germany). The volume of the primary tumor was measured using a Vernier caliper and calculated using the following formula: π/6 × (L ×W× W), where L is the tumor at its longest, and W is the tumor at its shortest. The primary sites were analyzed by immunohistochemical staining followed by microscopic examination. The numbers of mouse CD8^+^ and CD4^+^ cells in 2 different HPFs per sample were quantified manually. The stromal expression of CD11c was assessed and divided into two groups (high and low) based on the intensity and area of expressions in the TME of murine PDAC.

### Murine *in vivo* antibodies and drug preparation

Mice were treated intraperitoneally with αPD-1 (RMP1-14, BioXcell, Lebanon, NH, USA; 200 μg per dose), αCTLA-4 (9H10, BioXcell; 200 μg per dose), and mC4BPA peptides consisting of 30 or 54 amino acids (Toray Research Center, Tokyo, Japan; 50 μg of 30 amino acids or 100 μg of 54 amino acids). Mouse IgG2a (Agilent; 200 μg) was used as an isotope control. All antibodies were endotoxin-free. Pharmaceutical grade Gem (Yakult Pharmaceutical, Tokyo, Japan) and nab-paclitaxel (Taiho Pharmaceutical, Tokyo, Japan) powders were purchased from Chiba University Hospital and resuspended in sterile normal saline at 40 mg/mL and 5 mg/mL, respectively. The drugs were administered by intraperitoneal injection at a dose of 120 mg/kg diluted in PBS.

### Statistical analysis

All statistical analyses were conducted using JMP® Pro, version 13.2.0 (SAS Institute Inc., Cary, NC, USA). Cumulative rates were calculated using the Kaplan-Meier method. The significance of differences in survival rate was analyzed by the log-rank test. Data are expressed as the mean ± standard deviation (SD) or SEM. Survival data were evaluated using univariate and multivariate Cox proportional regression analyses. Statistically significant differences were determined by Welch’s *t*-test, chi-square test, and Mann–Whitney–Wilcoxon test. *P* < 0.05 was considered statistically significant in all analyses. The asterisk (*) indicates a significant value. Each experiment was repeated at least three times.

## Results

### Stromal C4BPA expression was positively correlated with TILs and is associated with a favorable prognosis in PDAC patients

**Fig. 1:**
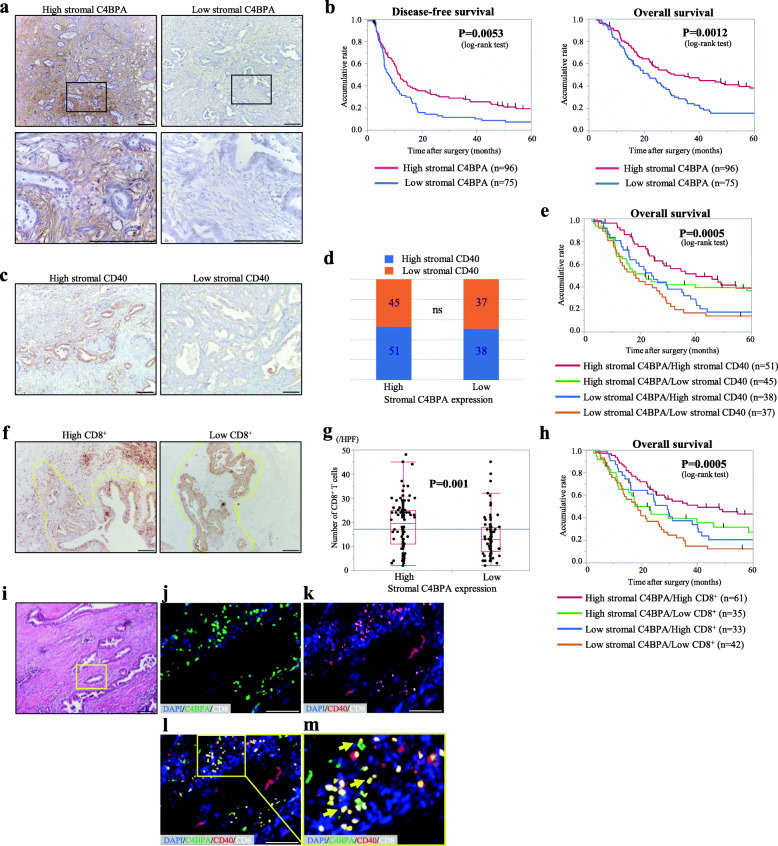
Immunohistochemical analysis of C4BPA, CD40, and CD8 expression in resected human PDAC samples. **a** C4BPA staining patterns in primary PDAC tissues (*n*=171) were categorized into high (left) and low stromal C4BPA expression (right) based on the intensity criterion. The boxed area is magnified in the lower panel. Original magnification: upper panels ×40, lower panels ×200. **b** Kaplan-Meier analyses for DFS and OS of PDAC patients based on stromal C4BPA expression. The high stromal C4BPA group presented significantly longer DFS (*P*=0.0053, log-rank test) and OS (*P*=0.0012, log-rank test) than did the low stromal C4BPA group after curative surgery. **c** CD40 staining patterns in primary PDAC tissue were categorized into high (left) and low stromal CD40 expression (right). Original magnification: ×40. **d** Correlation of C4BPA and CD40 expression in the stroma of human PDAC tissues. **e** Kaplan-Meier analyses for OS of PDAC patients based on stromal C4BPA and CD40 expression. Patients with high stromal C4BPA and CD40 presented significantly longer OS (*P*=0.0005, log-rank test) than did other patients. **f** CD8^+^ T cell staining patterns in primary PDAC tissue were categorized into high (left) or low CD8^+^ expression (right). Original magnification: ×40. **g** Correlation of C4BPA and CD8 expression in stroma of human PDAC tissues (*P*=0.001, Mann–Whitney–Wilcoxon test). **h** Kaplan-Meier analyses for OS of PDAC patients based on CD8 expression. Patients with high stromal C4BPA/CD8^+^ presented significantly longer OS (*P*=0.0005, log-rank test) than did other patients. **i**–**m** Immunofluorescence analysis of CD8^+^ TILs in human PDAC tissues. **i** H&E staining and (**j**–**m**) quadruple immunofluorescence staining for C4BPA (green), CD40 (red), CD8 (white), and DAPI (blue). Boxed regions highlight regions where stromal C4BPA is coexpressed with CD40 and CD8 in the TME of primary PDAC tissues. Yellow arrows indicate areas where enrichment of stromal CD8^+^ cells expressing C4BPA was observed. Magnification: ×100 for c and f. Bar, 50 μm. P-values are indicated in the respective tables. ns, not significant.

To investigate C4BPA expression and localization in PDAC, immunohistochemical staining of resected human PDAC tissues was performed. C4BPA was not expressed in the PDAC tumor cells but was predominantly expressed in the stroma surrounding the PDAC cells (Fig. 1a). Based on the staining intensity of C4BPA in the stroma, PDAC samples were categorized into two groups, with 75 cases (43.9%) in the high stromal expression group and 97 cases (56.1%) in the low stromal expression group (Fig. 1a). However, there were no significant differences in various clinicopathological parameters between the high and low stromal C4BPA groups (Table 1). Kaplan-Meier analysis indicated that patients with high stromal C4BPA presented significantly longer disease-free survival (DFS) (*P*=0.0053) and overall survival (OS) than did those with low stromal C4BPA (*P*=0.0012) (Fig. 1b).

**Table 1:** Characteristics of PDAC patients in IHC analysis for stromal C4BPA expression

	C4BPA expression		*P* value
	High (*n*=96)	Low (*n*=75)	
Age (y.o. mean ± SD)	65.7±9.3	65.9±10.4	0.87
Gender (male/female)	51/45	48/27	0.15
Tumor location (head/body-tail)	67/30	56/19	0.50
Tumor volume (mm^3^ median (IQR))	6462 (4450,13124)	6283 (4450,13124)	0.18
UICC			
pT stage (pT3,4/pT1,2)	95/1	72/3	0.20
pN stage (pN1,2,3/pN0)	74/22	54/21	0.45
Curability (R1,2/R0)	36/60	34/41	0.30
Perioperative therapy			
Neoadjuvant therapy (+/−)	36/60	38/37	0.84
Adjuvant chemotherapy (+/−)	85/11	61/14	0.19
Recurrence site			
Local recurrence (+/−)	36/60	31/44	0.61
Hematogenous recurrence (+/−)	49/47	48/27	0.088

(chi-square test).

*SD* Standard deviation, *IQR* Interquartile range, *p* Pathological findings, *T* Primary tumor, *N* Regional lymph nodes

Next, as CD40 is the C4BPA receptor known to be expressed in various immunocompetent cells, we examined its expression pattern in the PDAC tissues. CD40 expression was observed in almost all PDAC cells and was observed in various quantities in the stroma of PDAC (Fig. 1c). Similar to C4BPA, stromal CD40 expression was divided into two groups based on the CD40 staining score in the PDAC stroma (high stromal CD40, *n*=89 [52.0%]; low stromal CD40, *n*=82 [48.0%]) (Fig. 1d, Supplementary Table 1). According to Kaplan-Meier analysis, the high stromal CD40 group also presented better prognosis than did the low stromal CD40 group (*P*=0.042) (Supplementary Fig. 1a). Notably, even though stromal CD40 expression was not correlated with stromal C4BPA expression in PDAC tissues (Fig. 1d), patients with both high stromal C4BPA and CD40 demonstrated a favorable outcome in PDAC (*P*=0.0005) (Fig. 1e).

One of the most crucial reactions of the host immune response against tumor cells is the accumulation of CD8^+^ T cells (CD8^+^ cells), which are considered TILs and enhance antitumor immunity. The number of CD8^+^ cells at the invasive front of the tumor was analyzed in resected human PDAC tissues by immunohistochemical staining (Fig. 1f, Supplementary Table 2). According to Kaplan-Meier analysis, the high CD8^+^ group showed better prognosis than that of the low CD8^+^ group (*P*=0.0013) (Supplementary Fig. 1b). Interestingly, stromal C4BPA expression strongly correlated with the number of CD8^+^ cells in primary PDAC (*P*=0.001) (Fig. 1g). Notably, Kaplan–Meier analysis showed that patients with high stromal C4BPA and CD8^+^ had a significantly longer OS than did those with lower expression (*P*=0.0005) (Fig. 1h). Additionally, according to Cox proportional hazard models, lymph node metastasis and low stromal C4BPA expression were independent factors in poor prognosis of patients with PDAC via multivariate analysis (Table 2). These data suggest that stromal C4BPA expression is positively correlated with TILs and that high stromal C4BPA is associated with a favorable prognosis in PDAC patients.

**Table 2:** Univariate and multivariate analyses for 5-year survival of patients

	*n*=171	Univariate analysis		Multivariate analysis	
		Hazard ratio (95%CI)	*P* value	Hazard ratio (95%CI)	*P* value
Age (year) (66≦/≧67)	(83/88)	1.54 (0.79-3.02)	0.19		
Gender (male/female)	(99/72)	1.17 (0.60-2.28)	0.63		
Histological grade (G3,4/G1,2)	(18/153)	0.78 (0. 27-2.15)	0.78		
Tumor volume (≧6335/6335<)	(82/89)	1.05 (0.54-2.05)	0.86		
pT3,4/pT1,2	(167/4)	2.55 (0.34-18.65)	0.36		
**pN (+/−)**	(128/43)	2.96 (1.43-6.14)	0.0036^*^	3.01 (1.37-6.59)	0.006^*^
R1,2/R0	(70/101)	2.12 (1.03-4.33)	0.034^*^	1.66 (0.78-3.54)	0.19
Neoadjuvant therapy (+/−)	(74/97)	1.02 (0.52-2.00)	0.944		
Adjuvant chemotherapy (+/−)	(146/25)	0.42 (0.13-1.31)	0.11		
**Stromal C4BPA (Low/High)**	(75/96)	2.50 (1.22-5.10)	0.0096^*^	2.54 (1.18-5.48)	0.017^*^
Stromal CD40 (Low/High)	(82/89)	1.05 (0.54-2.05)	0.86		
CD8 expression (Low/High)	(77/94)	2.06 (1.02-4.13)	0.037^*^	1.67 (0.80-3.50)	0.16

Cox’s proportional hazard model

*p* Pathological findings, *T* Primary tumor, *N* Regional lymph nodes, *R* Surgical curability, *CI* Confidence interval, *: significant value

### C4BPA is coexpressed with CD8^+^ T cells in the stroma surrounding PDAC cells

To validate the localization of C4BPA, CD40, and TILs in PDAC tissues, immunofluorescence staining was performed in resected PDAC tissues. Quadruple staining indicated that C4BPA, CD40, and CD8^+^ are coexpressed in the stroma surrounding PDAC cells (Fig. 1i–m). Taken together, these data suggest that the activation of the C4BPA-CD40 axis may facilitate an antitumor response via CD8^+^ T cell accumulation in the TME of PDAC. Cancer-associated fibroblasts (CAFs) are pathologically activated fibroblasts that are abundant in the stroma of PDAC. CAFs are a highly secretory cell population that plays key roles in constructing the TME and modulating its functions during tumor progression. We investigated whether C4BPA was coexpressed in CAFs expressing α-smooth muscle actin (α-SMA). Immunofluorescence staining indicated that C4BPA did not merge with α-SMA in the stroma of PDAC (Supplementary Fig. 1c). This result suggests that C4BPA is not secreted by CAFs in the TME of PDAC.

### C4BPA stimulation increases the number of both CD4^+^ and CD8^+^ T cells in PBMCs

**Fig. 2:**
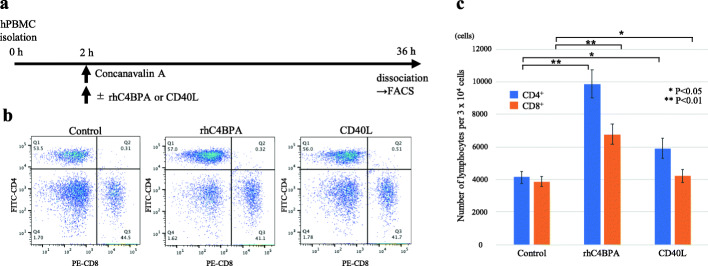
C4BPA stimulation in human PBMCs. **a** PBMC culture protocol. **b** Representative scatter plots of CD4^+^ and CD8^+^ T cells in the control, rhC4BPA stimulation, and CD40L stimulation groups, as analyzed by flow cytometry. **c** Number of CD4^+^ and CD8^+^ cells in each group. After PBMCs were stimulated with rhC4BPA and CD40L, the number of both CD4^+^ and CD8^+^ T cells in these two stimulation groups significantly increased compared with that in the control group (rhC4BPA stimulation: **P < 0.01, CD40L stimulation: *P < 0.05, Welch’s *t*-test). Results are represented as the mean ± SD. Each experiment was performed at least three times, independently.

Previous reports have described that C4BPA directly binds to CD40 on dendritic cells, B cells, and macrophages, resulting in CD4^+^ and CD8^+^ T cell activation [30–33]. To confirm the effect of C4BPA on CD4^+^ and CD8^+^ T cell proliferation, PBMCs including dendritic cells, B cells, T cells, and macrophages were analyzed using flow cytometry (Fig. 2a). PBMCs were stimulated with concanavalin A and rhC4BPA or human CD40L, which was used as positive control. The number of both CD4^+^ and CD8^+^ T cells was significantly increased in the rhC4BPA group compared with that in the control and CD40L groups (Fig. 2b, c). These results indicated that C4BPA stimulation activates CD4^+^ and CD8^+^ T cell proliferation in PBMCs *in vitro*.

### C4BPA promotes cancer cell proliferation in CD40-expressing PDAC cells

**Fig. 3:**
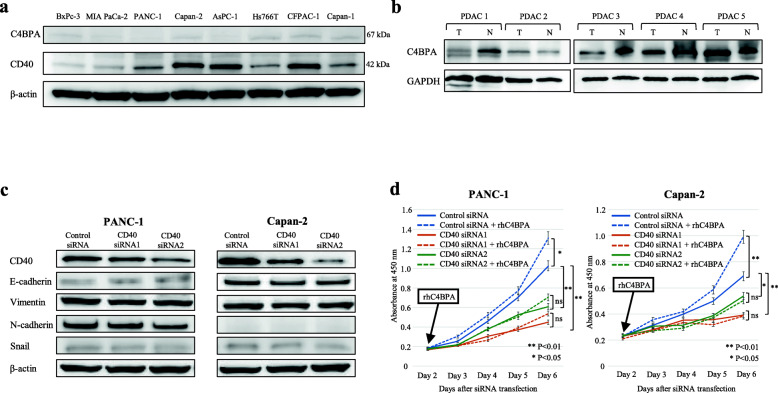
Differential expression of C4BPA and CD40 in human PDAC cell lines and PDAC tissues. **a** Western blot analysis of C4BPA and CD40 expression in various human PDAC cell lines, including cell lines of primary PDAC (BxPC-3, MIA PaCa-2, PANC-1, Capan-2), metastatic ascites (AsPC-1), lymph node metastasis (Hs766T), and PDAC liver metastases (CFPAC-1, Capan-1). **b** Western blot analysis of C4BPA protein expression in resected PDAC tissues. T: tumor tissues, N: adjacent normal pancreatic tissues. **c** CD40 expression was decreased by the CD40-specific siRNAs in PANC-1 and Capan-2 cells. The expressions of representative EMT markers, E-cadherin, Vimentin, N-cadherin, and Snail did not alter in CD40-knockdown PANC-1 and Capan-2 cells. **d** CD40 knockdown impairs cell proliferation in PDAC cells. Cell proliferation was significantly decreased in CD40-knockdown PANC-1 and Capan-2 cells (*P < 0.05, **P < 0.01, Welch’s *t*-test). rhC4BPA stimulation (at day 2) significantly increased cell proliferation in cell lines highly expressing CD40, including PANC-1 (*P < 0.05, Welch’s *t*-test) and Capan-2 (**P < 0.01, Welch’s *t*-test). However, proliferation in CD40 knockdown cells was unchanged (not significant, Welch’s *t*-test). Experiments were performed three times independently. Error bars represent the SD. ns, not significant.

In the immunohistochemical analysis, CD40 was observed not only in stromal immunocompetent cells, but also in tumor cells of PDAC tissues. We hypothesized that C4BPA may also affect the tumor cell itself in PDAC. To clarify this hypothesis, we first confirmed C4BPA and CD40 protein expression in human PDAC cell lines. C4BPA was not expressed in human PDAC cell lines of primary and metastatic lesions or in the supernatant of cultured cells (Fig. 3a, Supplementary Fig 2a). In contrast, CD40 protein showed various amounts of expression in human PDAC cell lines (Fig. 3a). To validate the presence of C4BPA in the TME of PDAC, we extracted proteins from frozen human resected PDAC tissues. C4BPA was expressed in both PDAC lesions and adjacent normal pancreatic tissues (Fig. 3b). These data firmly support that C4BPA is expressed not in tumor cells, but in the TME of PDAC. Among all the human primary PDAC cell lines, PANC-1 and Capan-2, which are cells that highly express CD40, were used for further experiments.

To explore the relationship between CD40 and epithelial plasticity in PDAC cells, endogenous CD40 expression was suppressed by two CD40 specific siRNAs (Fig. 3c). The effect of CD40 knockdown was maintained until 6 days after the siRNA transfection (Supplementary Fig. 2b). CD40 knockdown did not affect the expressions of various representative epithelial or mesenchymal markers in PANC-1 or Capan-2 cells (Fig. 3c). This finding suggests that intrinsic CD40 expression is not involved in epithelial plasticity in PDAC cells. Next, to assess the *in vitro* effects of C4BPA on tumor cell proliferation that are exerted via CD40, we examined PDAC cell proliferation with CD40 knockdown. CD40 knockdown cells were obtained using CD40 siRNAs with or without rhC4BPA stimulation. The proliferation assays demonstrated that CD40 knockdown significantly decreased cell proliferation in PANC-1 and Capan-2 cells (Fig. 3d). Interestingly, rhC4BPA promoted cell proliferation in all cell lines that highly express CD40, whereas it did not alter the proliferation of CD40 knockdown cells. Additionally, rhC4BPA significantly increased cell proliferation in the high CD40 expression cell line CFPAC-1, while it did not increase proliferation in the low CD40 expression cell line MIA PaCa-2 (Supplementary Fig. 2c, d). These data revealed that C4BPA stimulation increases cell proliferation ability in CD40-expressing PDAC cells.

### Combining C4BPA with Gem cancelled proliferative status by facilitating chemosensitivity in PDAC cells

**Fig. 4:**
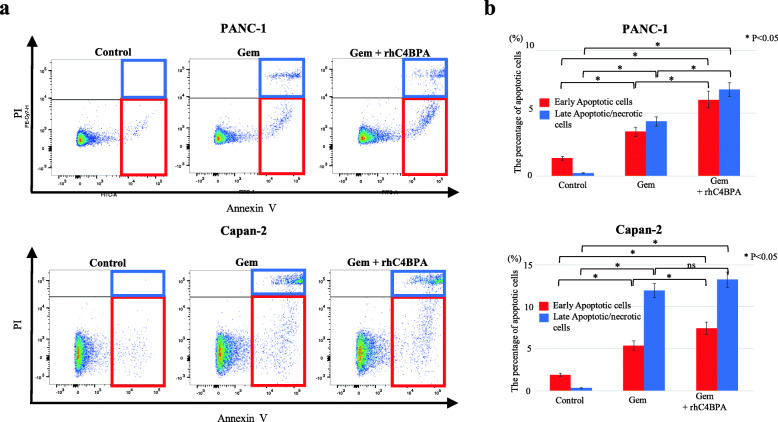
Enhanced apoptotic cell death in the combination of gemcitabine with C4BPA stimulation in human PDAC cells. PANC-1 (upper panels) and Capan-2 (lower panels) cells were treated with control, Gem, or Gem+rhC4BPA for the apoptosis assay. **a** Analysis of apoptosis by flow cytometry was assessed by Annexin V/PI double-staining. **b** Comparative analysis of subpopulation of apoptotic cells in PDAC cells treated with control, Gem, or Gem+rhC4BPA. Red or blue box/column indicates early apoptotic cells or late apoptotic/necrotic cells, respectively. Experiments were performed three times independently. Error bars represent the SD. ns, not significant.

The classical chemotherapeutic agent Gem was designed to impair the proliferative capacity of cancer cells by inhibiting DNA synthesis. Cancer cells with hyperproliferative capacity possibly show vulnerability to chemotherapy before acquiring chemoresistance. We examined whether C4BPA stimulation induces more sensitivity to increase the efficacy of Gem in PDAC cells. Despite an increase in cell proliferation due to C4BPA stimulation, the cytotoxic efficacy of low-dose Gem was increased, offsetting the increase in cell proliferation by C4BPA in PANC-1 and Capan-2 cells (Supplementary Fig. 3). Apoptosis is one of the major mechanisms for programmed cell death. Therefore, we next assessed whether apoptotic cell death is a canonical mechanism for increasing cytotoxicity by combining Gem and rhC4BPA. C4BPA stimulation significantly increased the number of early apoptotic cells, as demonstrated by annexin V/PI staining in PANC-1 and Capan-2 cells (Fig. 4a, b). These results suggest that C4BPA increases Gem sensitivity by driving apoptotic cell death, functioning as a chemosensitizing agent in CD40-expressing PDAC cells.

### Mouse C4BPA peptide increases murine CD4^+^ and CD8^+^ T cell proliferation

**Fig. 5:**
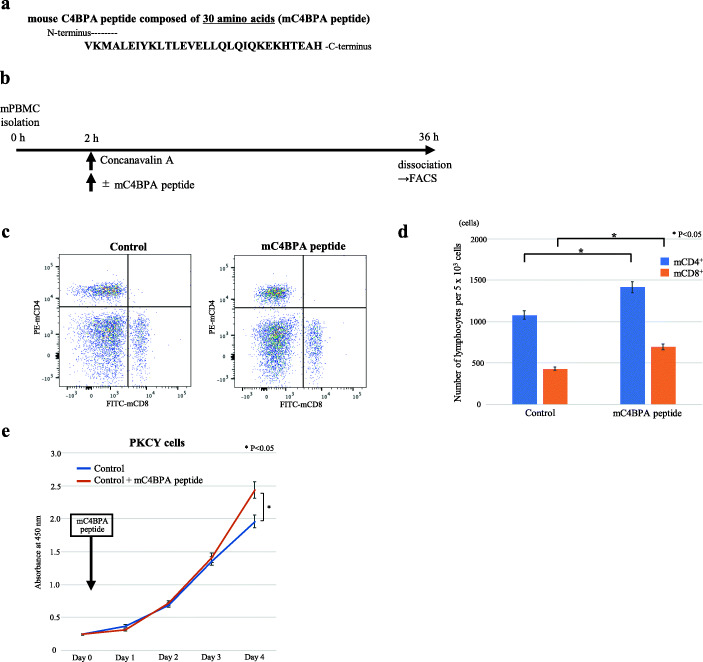
mC4BPA stimulation in mPBMCs and murine PDAC cells. **a** The mC4BPA peptide is composed of 30 amino acids from the C-terminus of mC4BPA. **b** mPBMC culture protocol. **c** Representative scatter plots of mCD4^+^ and mCD8^+^ T cells in the control and mC4BPA peptide stimulation groups, analyzed using flow cytometry. **d** Number of mCD4^+^ and mCD8^+^ cells on each group. After mPBMCs were stimulated with mC4BPA peptide, the numbers of both mCD4^+^ and mCD8^+^ T cells were significantly increased compared with those in the control group (*P < 0.05, Welch’s *t*-test). **e** mC4BPA peptide stimulation increased cell proliferation in PKCY cells compared with that in control cells (*P < 0.05, Welch’s *t*-test). Results are represented as the mean ± SD. Each experiment was performed at least three times.

C4BP consists of seven α-chain subunits and one β-chain. Structurally, heptameric C4BPA oligomerizes at the C-terminus, which contains intermolecular disulfide bridges to form the core complex [34]. A recent report described that the C-terminus of C4BPA mediates its interaction with CD40 [35]. Furthermore, the C-terminus domain of the alpha chain of mouse C4BP (mC4BPA) can act as an adjuvant to enhance the immune response in mice [36]. Considering that the C-terminal C4BPA binds to CD40, we designed two mC4BPA peptides composed of 30 amino acids (mC4BPA peptide) (Fig. 5a) and 54 amino acids (named as mC4BPA peptide [54]) from the C-terminus of mC4BPA (Supplementary Fig 4a). To conduct functional analyses *in vitro*, we assessed whether mC4BPA peptide activates cellular proliferation in murine cells as well as in human cells. We initially examined mC4BPA expression in murine pancreatic neoplastic cells. Similar to human PDAC cell lines, mC4BPA expression was not observed in all murine cells (Supplementary Fig. 4b). Consistent with the results observed in humans, flow cytometry revealed that the mC4BPA peptide successfully facilitated proliferation in both mCD4^+^ and mCD8^+^ T cells (Fig. 5b–d). Additionally, mouse PDAC cells also significantly increased cell proliferation 4 days after stimulation with mC4BPA peptide (Fig. 5e). The results of these experiments using mC4BPA peptide [54] were similar to those observed by stimulation with the mC4BPA peptide composed of 30 amino acids (Supplementary Fig 4c–e). These results revealed that the mC4BPA peptide increases the number of T lymphocytes in mice *in vitro*. Thus, we selected the mC4BPA peptide composed of 30 amino acids for further *in vivo* experiments.

### Mouse C4BPA peptide induces CD8^+^ T cell accumulation around PDAC tumors in an orthotopic transplantation model

**Fig. 6:**
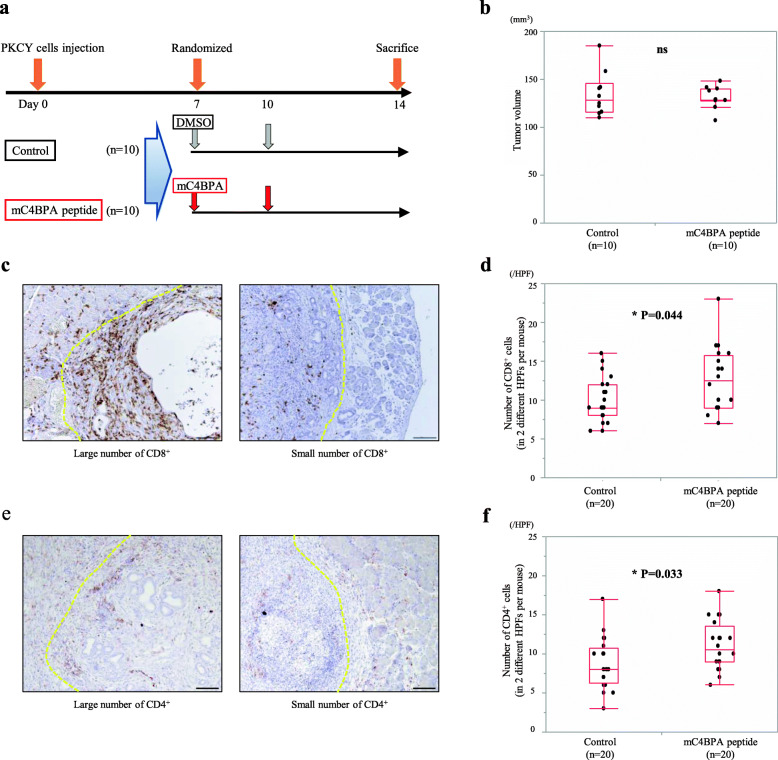
mC4BPA stimulation for the accumulation of CD8^+^ and CD4^+^ T cells in mPDAC tumors. **a** Experimental design of the *in vivo* experiment using PKCY cells in the orthotopic transplantation model. DMSO (control) and mC4BPA peptide were administered by intraperitoneal injection. **b** Pancreatic tumor volumes are depicted for the two groups in box-plot histograms. Each volume represents the mean ± SEM. **c** The representative figures of CD8^+^ T cells in the peripheral of PDAC tumors (left panel; large number of CD8^+^ T cells, right panel; small number of CD8^+^ T cells). Bar, 50 μm. **d** Number of CD8^+^ cells counting in 2 different high-power fields per mouse are depicted for the two groups in box-plot histograms (Control vs. mC4BPA peptide: *P=0.044, Mann–Whitney–Wilcoxon test). **e** The representative figures of CD4^+^ T cells in the peripheral of PDAC tumors (left panel; large number of CD4^+^ T cells, right panel; small number of CD4^+^ T cells). Bar, 50 μm. **f** Number of CD4^+^ T cells counting in 2 different high-power fields per mouse are depicted for the two groups in box-plot histograms (Control vs. mC4BPA peptide: *P=0.033, Mann–Whitney–Wilcoxon test).

Based on the *in vitro* findings, we conducted orthotopic transplantation of PDAC cells to assess the increase and recruitment of T lymphocytes in an *in vivo* experiment. After randomization at 7 days after cancer cell injection, experimental mice were treated with mC4BPA peptide twice by intraperitoneal injection (Fig. 6a). Compared to the control group, the tumor volume and the body weight showed no difference in the mC4BPA peptide group at 14 days after cell injection (Fig. 6b, Supplementary Fig 5a). Importantly, the number of CD8^+^ cells and CD4^+^ cells surrounding the PDAC tumor in the mC4BPA group were significantly higher than those in the control group (CD8^+^, *P*=0.044) (Fig. 6c, d), (CD4^+^, *P*=0.033) (Fig. 6e, f). Furthermore, the stromal expression of CD11c which is a typical mouse dendritic cell marker, was divided into two groups based on the staining intensity in the mouse PDAC stroma, the rate of high stromal CD11c (70%) was higher in the mC4BPA group compared with the control group (40%) (Supplementary Fig 5b–c). These findings implicated that mC4BPA acts on dendritic cells and fosters the accumulation of CD8^+^ T cells, resulting in the induction of antitumor immunogenicity in the TME of the PDAC tumor.

### C4BPA is recruited to the surrounding PDAC with TILs, showing T cell antitumor immunogenicity in *in vivo* models

**Fig. 7:**
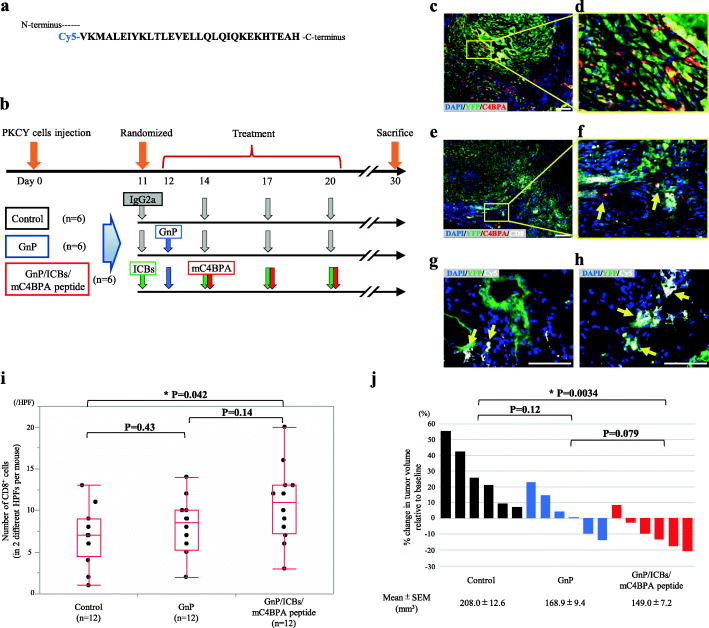
Administered mC4BPA peptide reaches PDAC stroma and functions *in vivo*. **a** The mC4BPA peptide, which is composed of 30 amino acids from the C-terminus of mC4BPA, was conjugated with Cyanine 5 (Cy5) at the N-terminus. **b** Experimental design of the randomized trial using PKCY cells in the orthotopic transplantation model. Mouse IgG2a antibody (Control); Gem and nab-paclitaxel (GnP); the combination of GnP and ICBs; and the combination of GnP, ICBs, and mC4BPA peptide were administered by intraperitoneal injection. **c**-**h** Immunofluorescence staining for representative pancreatic tumors in orthotopic transplantation models. (c, d) Immunofluorescence staining for DAPI, YFP, and mC4BPA in the GnP group; (e, f) for DAPI, YFP, mC4BPA, and CD8 in the GnP group; and (g, h) for DAPI, YFP, Cy5 (mC4BPA peptide) in the GnP/ICBs/mC4BPA peptide group. Yellow arrows show binding of the respective, fluorescence dye-labeled antibodies. Bar, 50 μm. **i** Number of CD8^+^ T cells counting in 2 different HPFs per mouse are depicted for treatment groups in box-plot histograms. (Control vs. GnP: *P*=0.43, Control vs. GnP/ICBs/mC4BPA peptide: *P=0.042, GnP vs. GnP/ICBs/mC4BPA peptide: *P*=0.14, Mann–Whitney–Wilcoxon test). **j** Waterfall plot indicating percent change in tumor volume after therapy compared with the tumor volume at baseline. (Control vs. GnP: *P*=0.12, Control vs. GnP/ICBs/mC4BPA peptide: *P=0.0034, GnP vs. GnP/ICBs/mC4BPA peptide: *P*=0.079, chi-square test). Each volume represents the mean ± SEM in the below box.

Considering the increase of cytotoxic effects induced by combining Gem with C4BPA, we conducted a preclinical trial to confirm the efficacy of Gem plus nab-paclitaxel (GnP), dual immune checkpoint blockades [ICBs; agonistic programmed cell death-1 (αPD-1) and agonistic cytotoxic T-lymphocyte-associated antigen-4 (αCTLA-4)], and mC4BPA peptide in the orthotopic transplantation model. To visualize the capture and localization of the extrinsic mC4BPA peptide in the TME of the PDAC tumor, we conjugated carboxylic acid 5 (Cy5) at the N-terminus of the mC4BPA peptide (Fig. 7a). After randomization, experimental mice were treated with standard chemotherapy, GnP, dual ICBs, and mC4BPA peptide (Fig. 7b, Supplementary Fig 6a). Immunofluorescence staining revealed that mC4BPA was abundantly expressed (Fig. 7c, d) and coexpressed with CD8^+^ T cells (Fig. 7e, f) in the stroma surrounding YFP-positive cancer cells. Notably, intraperitoneally injected mC4BPA peptides conjugated with Cy5 were observed in the periphery of PDAC lesions (Fig. 7g, h). Among these three experimental groups, the numbers of CD8^+^ cells in the GnP/ICBs/mC4BPA groups were greater than those in the control group (*P*=0.042) (Fig. 7i). Additionally, the numbers of CD4^+^ cells in the GnP/ICBs/mC4BPA group were significantly higher than those in the control group (*P*=0.048) (Supplementary Fig. 6b), and the rate of high stromal CD11c in the GnP/ICBs/mC4BPA group was the highest among the three groups (Supplementary Fig. 6c). These data suggest that mC4BPA facilitates the arrangement of anti-tumor immunogenicity and is accumulated to recruit cytotoxic CD8^+^ T cells and into the surrounding PDAC cells. Finally, we assessed and compared the antitumor efficacy of these experimental groups. Based on a comparison with the mean tumor volume (defined as the baseline) in mice that were sacrificed at 11 days after cancer cell injection, the antitumor effects of GnP/ICBs/mC4BPA peptide treatment group were evident by observing the induction of a greater number of tumor regressions among the three groups (Fig. 7j, Supplementary Fig. 6d). These results suggest that combination with classical chemotherapy, ICBs, and the mC4BPA peptide treatment impairs PDAC tumor volume in a mouse model of orthotopic transplantation.

## Discussion

**Fig. 8:**
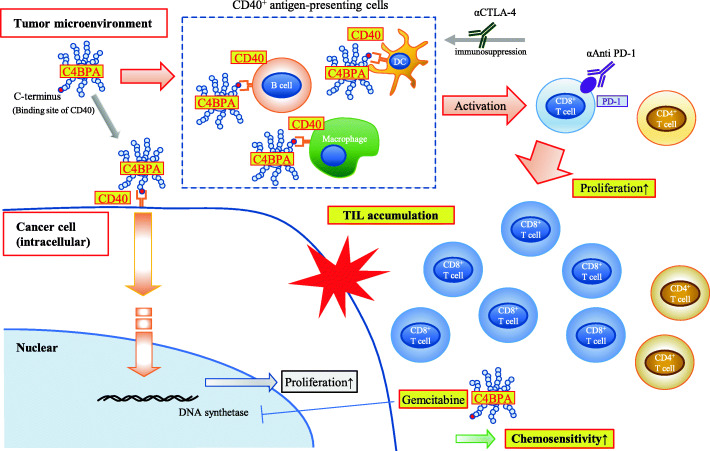
The schema of C4BPA functions in the tumor microenvironment of PDAC.

In this study, we demonstrate that C4BPA promotes CD8^+^ cell proliferation, resulting in the recruitment of TILs into the TME of PDAC by activating CD40 and enhancing T cell-mediated antitumor immunity in PDAC. Furthermore, C4BPA increases the chemosensitivity of Gem by inducing apoptotic programmed cancer cell death (Fig. 8). High stromal C4BPA and CD40 expression is associated with good clinical outcomes in patients with PDAC after surgery. To our knowledge, this is the first study to show that the C4BPA-CD40 axis fosters the activation of CD8^+^ TILs to facilitate the antitumor effect, resulting in better prognosis in solid tumors.

The TME is thought to greatly influence cancer growth and spread, thus impacting the clinical outcome of cancer patients. The majority of PDAC displays low T cell immunogenic features with poor TILs in the TME, indicating an “immuno-escape” mechanism for evading the host immune response [37]. Imbalanced complement activation in the TME blocks the release of immunocompetent cytokines by the antitumor CD8^+^ T cell responses. The roles of complement factors in cancer progression vary across cancer types, as they can promote or suppress tumor growth depending on the context [38]. Recent evidence suggests that components of the complement cascade function in cancer cell proliferation or survival. A previous study has provided evidence that local complement activation of C3a and C5a in the TME results in an immunosuppressive response to melanoma by inhibiting CD8^+^ TIL function [39].

C4BP is primarily known as a circulating soluble inhibitor of the classical complement pathways; it physiologically regulates inflammation caused by bacterial infections [40]. Contrary to the cancer-promoting functions of complement in tumors, we demonstrate that C4BPA mediates the recruitment of CD8^+^ TILs, presenting cancer-suppressive functions in the TME of PDAC. In this study, we demonstrated that C4BPA increases the cell proliferation ability of CD4^+^ and CD8^+^ T cells and PDAC cells in CD40-expressing PDAC cells. This finding suggests that C4BPA stimulation shows “a double-edged sword” effect for PDAC treatment; however, C4BPA stimulation increases Gem chemosensitivity, offsetting the increase of cell proliferation by inducing apoptosis in PDAC cells.

The loss of APCs surrounding the cancer cells in the stroma is one of the critical mechanisms that allow cancer cells to evade CD8^+^ T cell infiltration in PDAC. The ‘licensing’ of APC, in particular dendritic cells, results in up-regulation of membrane co-stimulatory molecules and the production of pro-inflammatory cytokines [41]. In that case, CD40 is closely involved in the functional maturation of APC and consequently the activation of antigen-specific T cells [42]. Recent evidence suggests that CD40 is a robust target for chemoimmunotherapy in PDAC. In mice, there is overwhelming evidence that CD40 activation enables a vaccination effect and the concomitant expansion of antigen-specific CD8^+^ T cells, which is dependent on CD40-expressing dendritic cells [43]. Indeed, our *in vivo* study implicated that C4BPA stimulation increases dendritic cells in the stroma of PDAC. Agonistic CD40 antibody (αCD40) has been developed as an immunotherapeutic agonist to activate T cell priming in PDAC [44]. Although tumor regressions were observed with agonistic αCD40 alone in a clinical trial of patients with metastatic PDAC, durable tumor regressions were not observed in the PDAC patients, with similar results obtained in mice [44, 45]. Mechanistically, the combination of αCD40/ICBs primed durable T cell responses, resulting in tumor regression and immunological memory [46]. Similarly, the present study demonstrates that the combination of GnP and ICBs/mC4BPA peptide treatment facilitates tumor regression in an orthotopic transplantation model of PDAC. Taken together, these reports suggest that mC4BPA peptides contribute to enhancing T cell antitumor immunity in a manner similar to that of αCD40 and could be an attractive model for treating PDAC in the future.

The limitations of this study are that CD40-expressing immunocompetent cells in which C4BPA directly activates CD8^+^ T cells, such as dendritic cells or B cells, were not assessed in the PBMC experiments. Long-term survival analysis was not conducted in the *in vivo* GEMM using combination chemotherapy and immunomodulatory therapy (ICBs/mC4BPA peptide). Further studies are warranted to determine the antitumor effect of C4BPA and the impact of immunomodulatory therapy.

## Conclusions

We demonstrate that the C4BPA-CD40 axis induces the activation of CD8^+^ TILs to exert an antitumor effect. Moreover, this axis can be targeted to improve the prognosis of PDAC patients. This study provides novel insight into complement-based combination therapy to further enhance T cell immunogenicity, presenting the possibility of its clinical application as a PDAC treatment strategy.

## Supplementary Information

**Additional file 1: Supplementary Table 1.** Characteristics of PDAC patients in IHC analysis for stromal CD40 expression. **Supplementary Table 2.** Characteristics of PDAC patients in IHC analysis for stromal CD40 expression. **Supplementary Table 3.** List of primary and secondary antibodies.

**Additional file 2: Supplementary information.** **Supplementary Fig. 1.** Clinical outcomes of patients with PDAC based on immunohistochemical analysis of CD40 and CD8 expression in resected human PDAC tissues. **Supplementary Fig. 2.** Recombinant human C4BPA stimulation increases proliferation in CD40 expressing PDAC cells. **Supplementary Fig. 3.** The cytotoxic efficacy in the combination of gemcitabine with C4BPA stimulation in human PDAC cells. **Supplementary Fig. 4.** mouse C4BPA expression and its peptide functions in mouse PDAC cells. **Supplementary Fig. 5.** Comparisons of mouse body weight and CD11c expression in the stroma of mPDAC tumors between mC4BPA peptide group and control group. **Supplementary Fig. 6.** Various parameters of the preclinical study.

**Additional file 3.**

## Data Availability

The datasets used and/or analyzed during the current study are available from the corresponding author on reasonable request.

## abbreviations

APCs: Antigen-presenting cells
α-SMA: α-Smooth muscle actin;
αCD40: Agonistic CD40
CAFs: Cancer-associated fibroblasts
C4BPA: C4b-binding protein α-chain
CD40L: CD40 ligand
DFS: Disease-free survival
DMEM: Dulbecco’s Modified Eagle Medium
FBS: Fetal bovine serum
H&E: Hematoxylin and eosin
HPF: High-power fields
hPBMCs: Human peripheral blood mononuclear cells
HRP: Horseradish peroxidase
ICB: Immune checkpoint blockade
mC4BPA: Mouse C4b-binding protein α-chain
mPBMCs: Murine peripheral blood mononuclear cells
OS: Overall survival
PBMCs: Peripheral blood mononuclear cells
PBS: Phosphate-buffered saline
PDAC: Pancreatic ductal adenocarcinoma
rhC4BPA: Recombinant human C4b-binding protein α-chain
RIPA: Radioimmunoprecipitation assay
SD: Standard deviation
SEM: Standard error of the mean
TIL: Tumor-infiltrating lymphocytes
TME: Tumor microenvironment
