# Supplementary material for: C4b-binding protein α-chain enhances antitumor immunity by facilitating the accumulation of tumor-infiltrating lymphocytes in the tumor microenvironment in pancreatic cancer
Source: J Exp Clin Cancer Res. 2021 Jun 24;40:212. doi: 10.1186/s13046-021-02019-0 (PMC8228942; doi:10.1186/s13046-021-02019-0)
Supplement: Supplementary file 1 — Additional file 1: Supplementary Table 1. Characteristics of PDAC patients in IHC analysis for stromal CD40 expression. Supplementary Table 2. Characteristics of PDAC patients in IHC analysis for stromal CD40 expression. Supplementary Table 3. List of primary and secondary antibodies. [file 13046_2021_2019_MOESM1_ESM.docx]

**Supplementary Table 1. Characteristics of PDAC patients in IHC analysis for stromal CD40 expression**

|  | CD40 expression | | P value |
| --- | --- | --- | --- |
|  | High (n=89) | Low (n=82) |  |
| Age (mean ± SD) | 66.7±9.7 | 64.9±9.6 | 0.22 |
| Gender (male/female) | 51/38 | 48/34 | 0.87 |
| Tumor location (head/body-tail) | 66/23 | 57/25 | 0.50 |
| Tumor volume (mm^3^ median) | 6446 | 6283 | 0.11 |
| UICC |  |  |  |
| pT stage (T4,3/T2,1) | 88/1 | 79/3 | 0.26 |
| pN stage (N3,2,1/N0) | 67/22 | 61/21 | 0.89 |
| Curability (R2,1/R0) | 31/58 | 39/43 | 0.09 |
| Perioperative therapy |  |  |  |
| Neoadjuvant therapy (+/−) | 39/50 | 35/47 | 0.88 |
| Adjuvant chemotherapy (+/−) | 72/17 | 74/8 | 0.08 |
| Recurrence site |  |  |  |
| Local recurrence (+/−) | 37/52 | 30/52 | 0.50 |
| Hematogenous recurrence (+/−) | 46/43 | 51/30 | 0.17 |

(chi-square test)

|  | CD8 expression | | P value |
| --- | --- | --- | --- |
|  | High (n=94) | Low (n=77) |  |
| Age (mean ± SD) | 65.8±9.6 | 65.8±9.8 | 0.99 |
| Gender (male/female) | 55/39 | 44/33 | 0.85 |
| Tumor location (head/body-tail) | 69/25 | 54/23 | 0.63 |
| Tumor volume (mm^3^ median) | 6633.5 | 6446.5 | 0.18 |
| UICC |  |  |  |
| pT stage (T3,4/T1,2) | 92/2 | 75/2 | 0.84 |
| pN stage (N1,2,3/N0) | 68/26 | 56/21 | 0.40 |
| Curability (R1,2/R0) | 36/58 | 34/43 | 0.43 |
| Perioperative therapy |  |  |  |
| Neoadjuvant therapy (+/−) | 44/50 | 30/47 | 0.30 |
| Adjuvant chemotherapy (+/−) | 84/10 | 62/15 | 0.080 |
| Recurrence site |  |  |  |
| Local recurrence (+/−) | 40/54 | 27/50 | 0.31 |
| Hematogenous recurrence (+/−) | 49/45 | 48/29 | 0.17 |

**Supplementary Table 2. Characteristics of PDAC patients in IHC analysis**

**for CD8 expression**

(chi-square test)

**Supplementary Table 3. List of primary and secondary antibodies**

| **Primary antibody** | **Sources** | **Dilution** |
| --- | --- | --- |
| Anti-C4BPA polyclonal antibody | Abcam, Cambridge, UK | WB 1:1000  IHC 1:400  IF 1:1000 |
| Anti-moues C4BPA polyclonal antibody | Cloud-Clone Corp., TX, USA | WB 1:1000  IF 1:1000 |
| Anti-CD40 monoclonal antibody | GeneTex, CA, USA | WB 1:500  IHC 1:200  IF 1:1000 |
| Anti-mouse CD40 monoclonal antibody | Santa Cruz Biotechnology, CA, USA | WB 1:1000 |
| Anti-CD8 alpha polyclonal antibody | Abcam, Cambridge, UK | IHC 1:200  IF 1:1000 |
| Anti-mouse CD8 alpha [D4W2Z] monoclonal antibody | Abcam, Cambridge, UK | IHC 1:200  IF 1:1000 |
| Anti-mouse CD4 [D7D2Z] monoclonal antibody | Cell Signaling Technology, MA, USA | IHC 1:200 |
| Anti-mouse CD11c [D1V9Y] monoclonal antibody | Cell Signaling Technology, MA, USA | IHC 1:200 |
| Anti-GFP polyclonal antibody | Abcam, Cambridge, UK | IF 1:400 |
| Anti-E-cadherin polyclonal antibody | Santa Cruz Biotechnology, CA, USA | WB 1:1000 |
| Anti-Vimentin polyclonal antibody | Cell Signaling Technology, MA, USA | WB 1:2000 |
| Anti-N-cadherin polyclonal antibody | Cell Signaling Technology, MA, USA | WB 1:2000 |
| Anti-Snail monoclonal antibody | Cell Signaling Technology, MA, USA | WB 1:2000 |
| PerCP anti-human CD3 [HIT3a] antibody | BioLegend, CA, USA | FCS 1:100 |
| FITC anti-human CD4 [A161A1] antibody | BioLegend, CA, USA | FCS 1:100 |
| PE anti-human CD8a [SK1] antibody | BioLegend, CA, USA | FCS 1:100 |
| PE anti-mouse CD4 [GK1.5] antibody | BioLegend, CA, USA | FCS 1:100 |
| FITC anti-mouse CD8a [53-6.7] antibody | BioLegend, CA, USA | FCS 1:100 |
| Anti-βactin monoclonal antibody | Cell Signaling Technology, MA, USA | WB 1:2000 |
| Anti-GAPDH polyclonal antibody | GeneTex Inc, CA, USA | WB 1:2000 |

| **Secondary antibody** | **Sources** | **Dilution** |
| --- | --- | --- |
| Anti-rabbit IgG horseradish peroxidase (HRP) antibody | Santa Cruz Biotechnology, CA, USA | WB 1:5000 |
| Anti-mouse IgGκ BP-HRP antibody | Santa Cruz Biotechnology, CA, USA | WB 1:5000 |
| Anti-hamster IgG-HRP antibody | Thermo Fisher Scientific, MA, USA | WB 1:5000 |
| Alexa Fluor 488 donkey anti-rabbit IgG antibody | Thermo Fisher Scientific, MA, USA | IF 1:500 |
| Alexa Fluor 488 goat anti-chicken IgY antibody | Abcam, Cambridge, UK | IF 1:500 |
| Alexa Fluor 546 goat anti-rat IgG antibody | Thermo Fisher Scientific, MA, USA | IF 1:500 |
| Alexa Fluor 647 donkey anti-rabbit IgG antibody | Abcam, Cambridge, UK | IF 1:500 |
| Alexa Fluor 647 goat anti-mouse IgG antibody | Thermo Fisher Scientific, MA, USA | IF 1:500 |

WB: western blotting, IHC: immunohistochemistry, IF: immunofluorescence staining,

FCS: flow cytometry standard
