# Supplementary material for: C4b-binding protein α-chain enhances antitumor immunity by facilitating the accumulation of tumor-infiltrating lymphocytes in the tumor microenvironment in pancreatic cancer
Source: J Exp Clin Cancer Res. 2021 Jun 24;40:212. doi: 10.1186/s13046-021-02019-0 (PMC8228942; doi:10.1186/s13046-021-02019-0)
Supplement: Supplementary file 2 — Additional file 2: Supplementary information. Supplementary Fig. 1. Clinical outcomes of patients with PDAC based on immunohistochemical analysis of CD40 and CD8 expression in resected human PDAC tissues. Supplementary Fig. 2. Recombinant human C4BPA stimulation increases proliferation in CD40 expressing PDAC cells. Supplementary Fig. 3. The cytotoxic efficacy in the combination of gemcitabine with C4BPA stimulation in human PDAC cells. Supplementary Fig. 4. mouse C4BPA expression and its peptide functions in mouse PDAC cells. Supplementary Fig. 5. Comparisons of mouse body weight and CD11c expression in the stroma of mPDAC tumors between mC4BPA peptide group and control group. Supplementary Fig. 6. Various parameters of the preclinical study. [file 13046_2021_2019_MOESM2_ESM.pptx]

## Slide 1
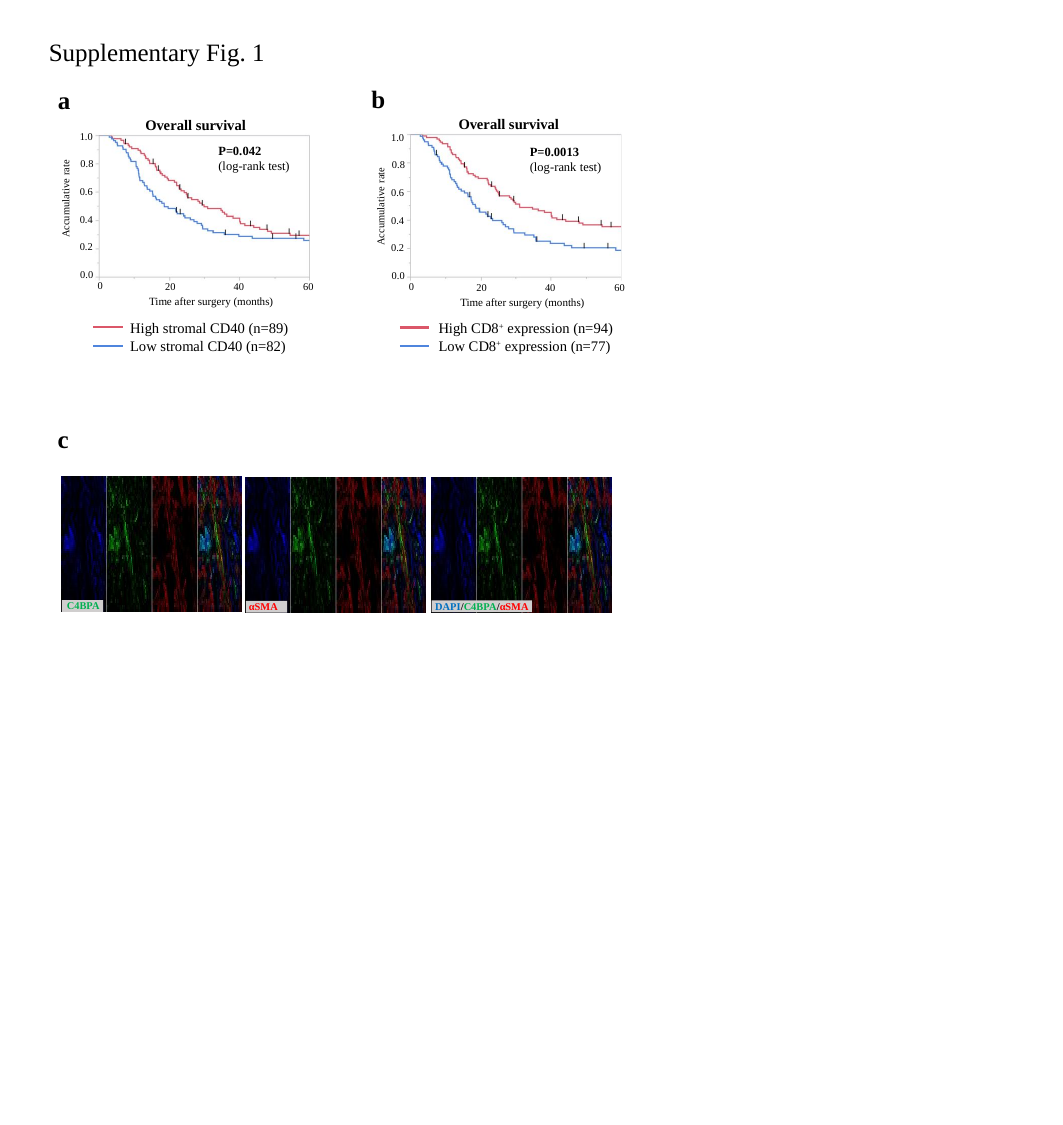

Supplementary Fig. 1
b
a
Overall survival
Overall survival
1.0
1.0
P=0.042
(log-rank test)
P=0.0013
(log-rank test)
0.8
0.8
0.6
0.6
Accumulative rate
Accumulative rate
0.4
0.4
0.2
0.2
0.0
0.0
0
60
20
40
0
60
20
40
 Time after surgery (months)
 Time after surgery (months)
High stromal CD40 (n=89)
Low stromal CD40 (n=82)
High CD8+ expression (n=94)
Low CD8+ expression (n=77)
c
C4BPA
αSMA
DAPI/C4BPA/αSMA

## Slide 2
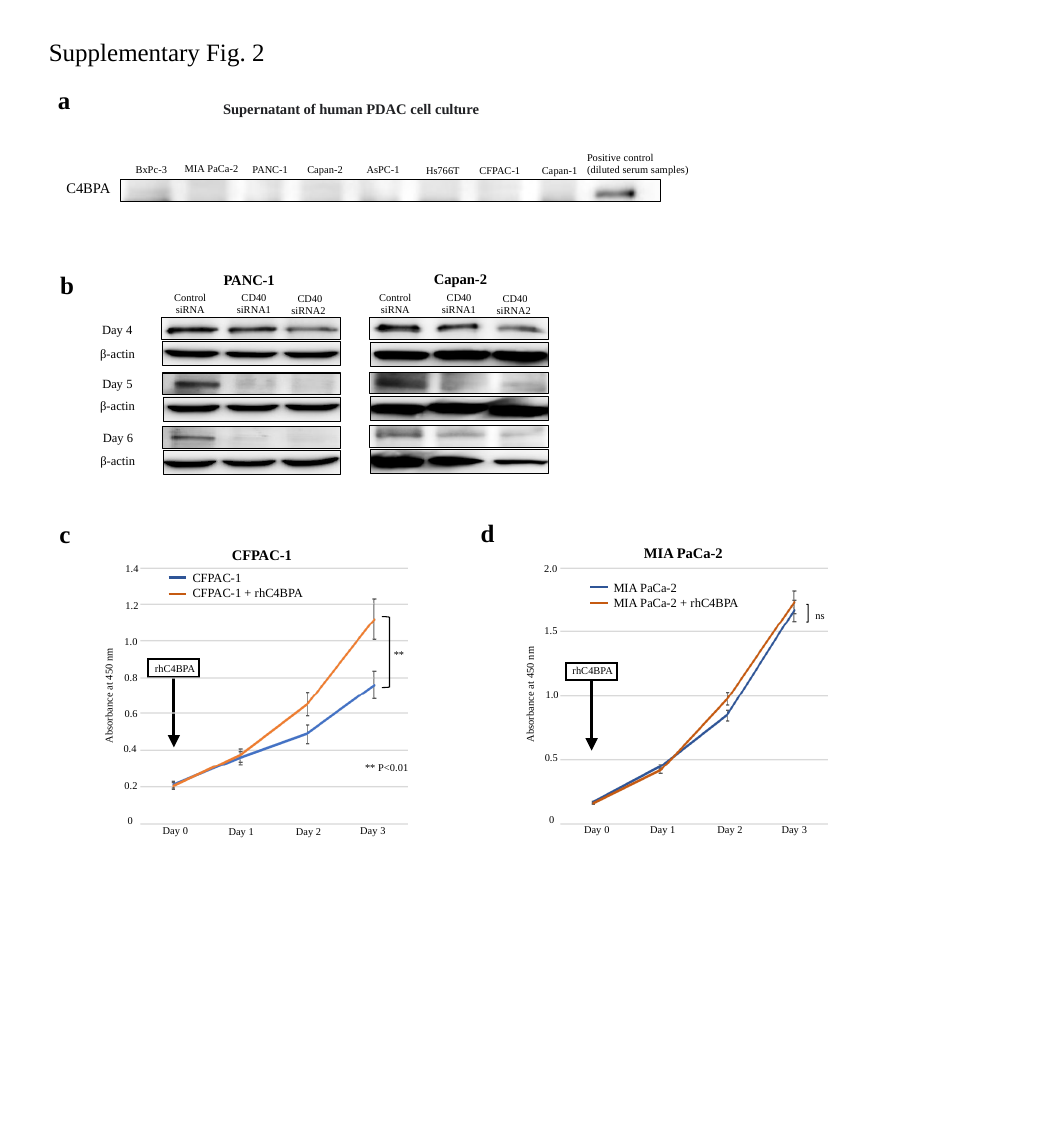

Supplementary Fig. 2
a
Supernatant of human PDAC cell culture
Positive control
(diluted serum samples)
MIA PaCa-2
Capan-2
PANC-1
BxPc-3
AsPC-1
Capan-1
CFPAC-1
Hs766T
C4BPA
b
Capan-2
PANC-1
CD40
siRNA1
CD40
siRNA1
Control
siRNA
Control
siRNA
CD40
siRNA2
CD40
siRNA2
Day 4
β-actin
Day 5
β-actin
Day 6
β-actin
d
c
MIA PaCa-2
CFPAC-1
2.0
1.4
CFPAC-1
CFPAC-1 + rhC4BPA
MIA PaCa-2
MIA PaCa-2 + rhC4BPA
1.2
ns
1.5
1.0
**
rhC4BPA
rhC4BPA
0.8
Absorbance at 450 nm
1.0
Absorbance at 450 nm
0.6
0.4
0.5
** P<0.01
0.2
0
0
Day 0
Day 3
Day 1
Day 2
Day 0
Day 3
Day 1
Day 2

## Slide 3
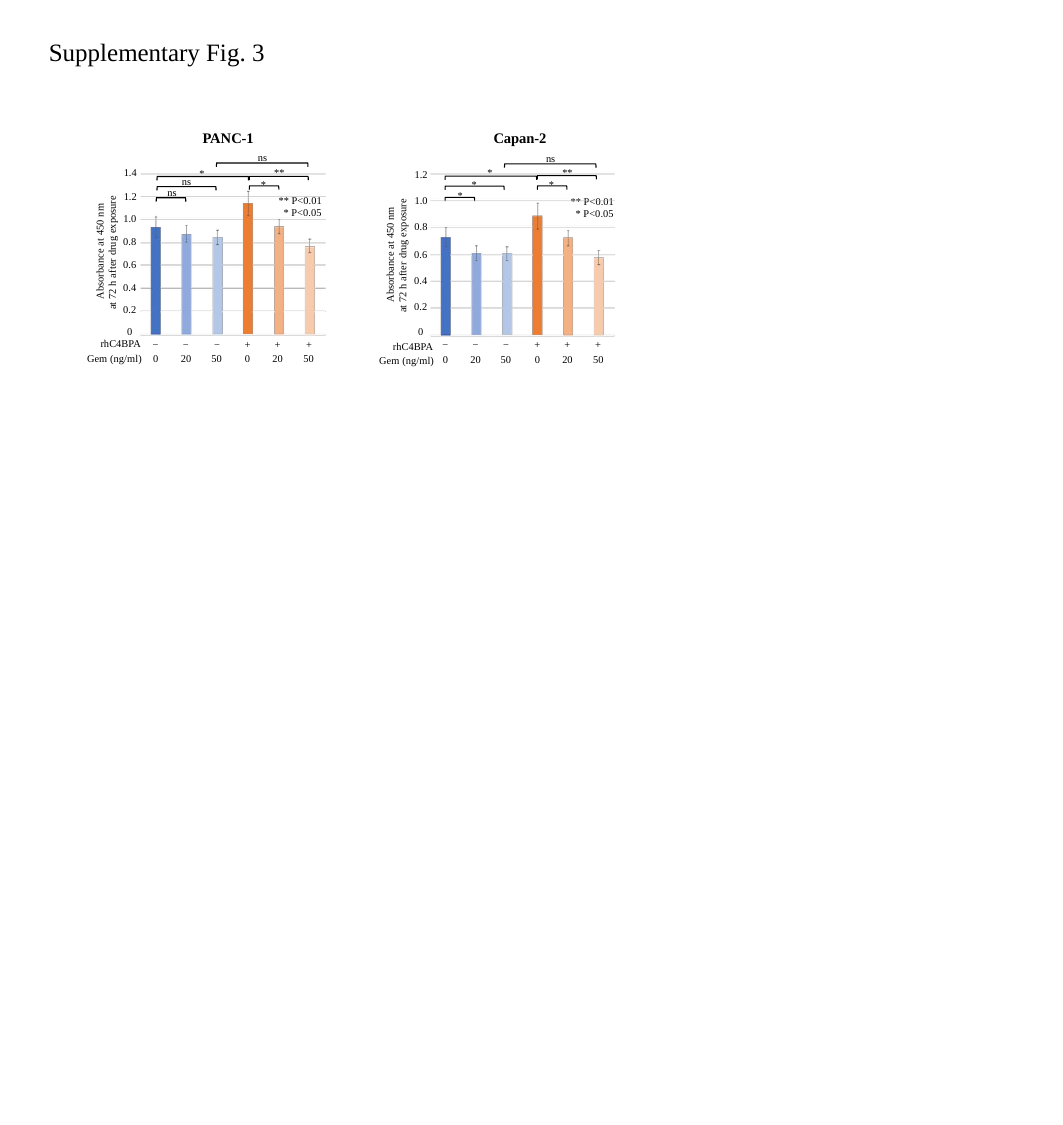

Supplementary Fig. 3
PANC-1
ns
**
1.4
*
ns
*
ns
1.2
** P<0.01
* P<0.05
1.0
0.8
Absorbance at 450 nm
at 72 h after drug exposure
0.6
0.4
0.2
0
−
+
−
−
+
+
rhC4BPA
0
0
20
50
20
50
Gem (ng/ml)
Capan-2
ns
*
**
1.2
*
*
*
1.0
** P<0.01
* P<0.05
0.8
Absorbance at 450 nm
at 72 h after drug exposure
0.6
0.4
0.2
0
−
+
−
−
+
+
rhC4BPA
0
0
20
50
20
50
Gem (ng/ml)

## Slide 4
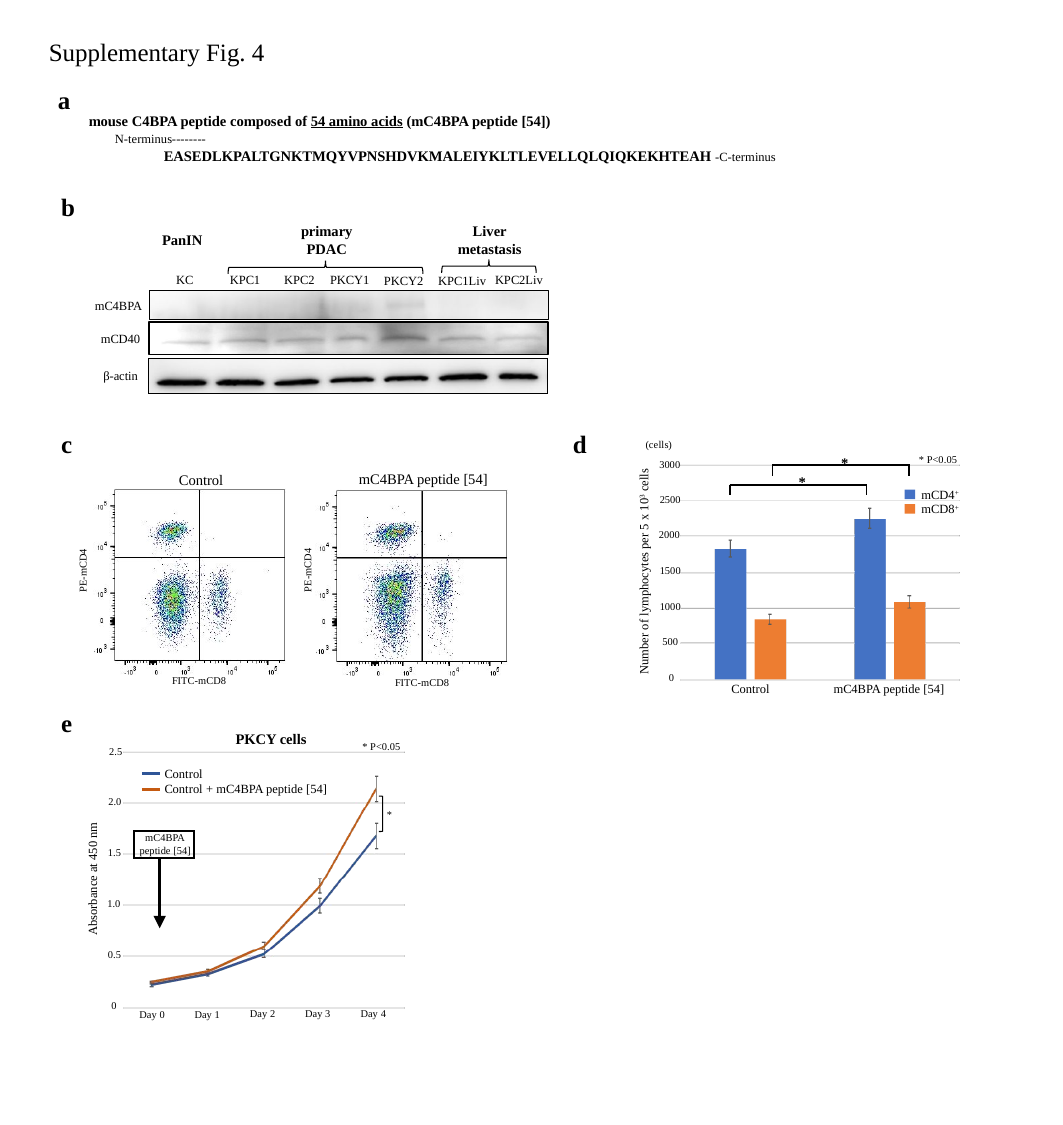

Supplementary Fig. 4
a
mouse C4BPA peptide composed of 54 amino acids (mC4BPA peptide [54])
 N-terminus--------
 EASEDLKPALTGNKTMQYVPNSHDVKMALEIYKLTLEVELLQLQIQKEKHTEAH -C-terminus
b
primary PDAC
Liver
metastasis
PanIN
KC
KPC1
KPC2
PKCY1
KPC2Liv
PKCY2
KPC1Liv
mC4BPA
mCD40
β-actin
(cells)
d
c
3000
* P<0.05
*
mC4BPA peptide [54]
Control
*
mCD4+
2500
mCD8+
2000
Number of lymphocytes per 5 x 10^3^ cells
1500
PE-mCD4
PE-mCD4
1000
500
0
FITC-mCD8
FITC-mCD8
Control
mC4BPA peptide [54]
e
PKCY cells
* P<0.05
2.5
Control
Control + mC4BPA peptide [54]
2.0
*
mC4BPA
peptide [54]
1.5
Absorbance at 450 nm
1.0
0.5
0
Day 4
Day 2
Day 3
Day 1
Day 0

## Slide 5
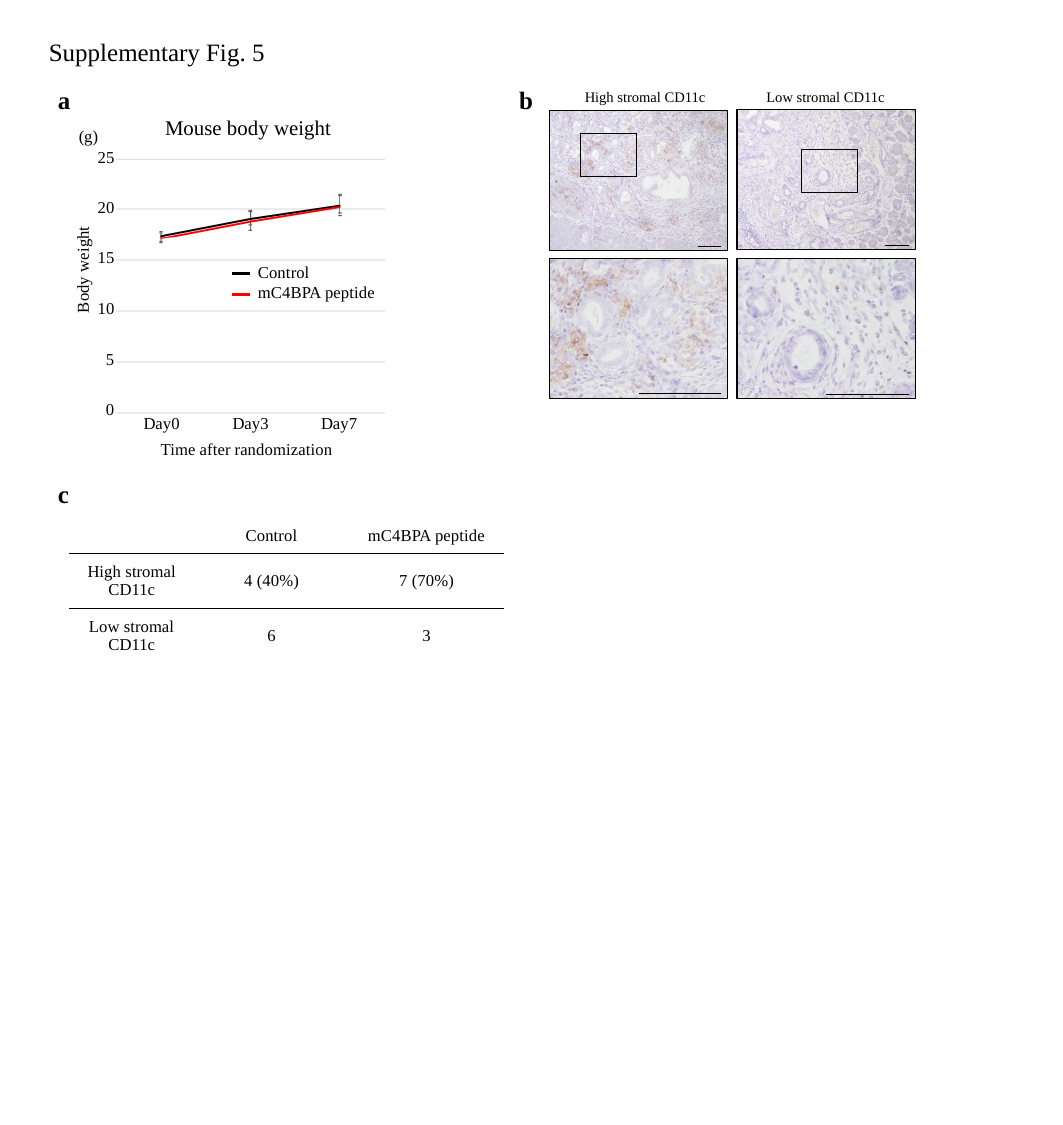

Supplementary Fig. 5
b
a
High stromal CD11c
Low stromal CD11c
Mouse body weight
(g)
25
20
15
Body weight
Control
mC4BPA peptide
10
5
0
Day7
Day0
Day3
Time after randomization
c
| | Control | mC4BPA peptide |
| --- | --- | --- |
| High stromal CD11c | 4 (40%) | 7 (70%) |
| Low stromal CD11c | 6 | 3 |

## Slide 6
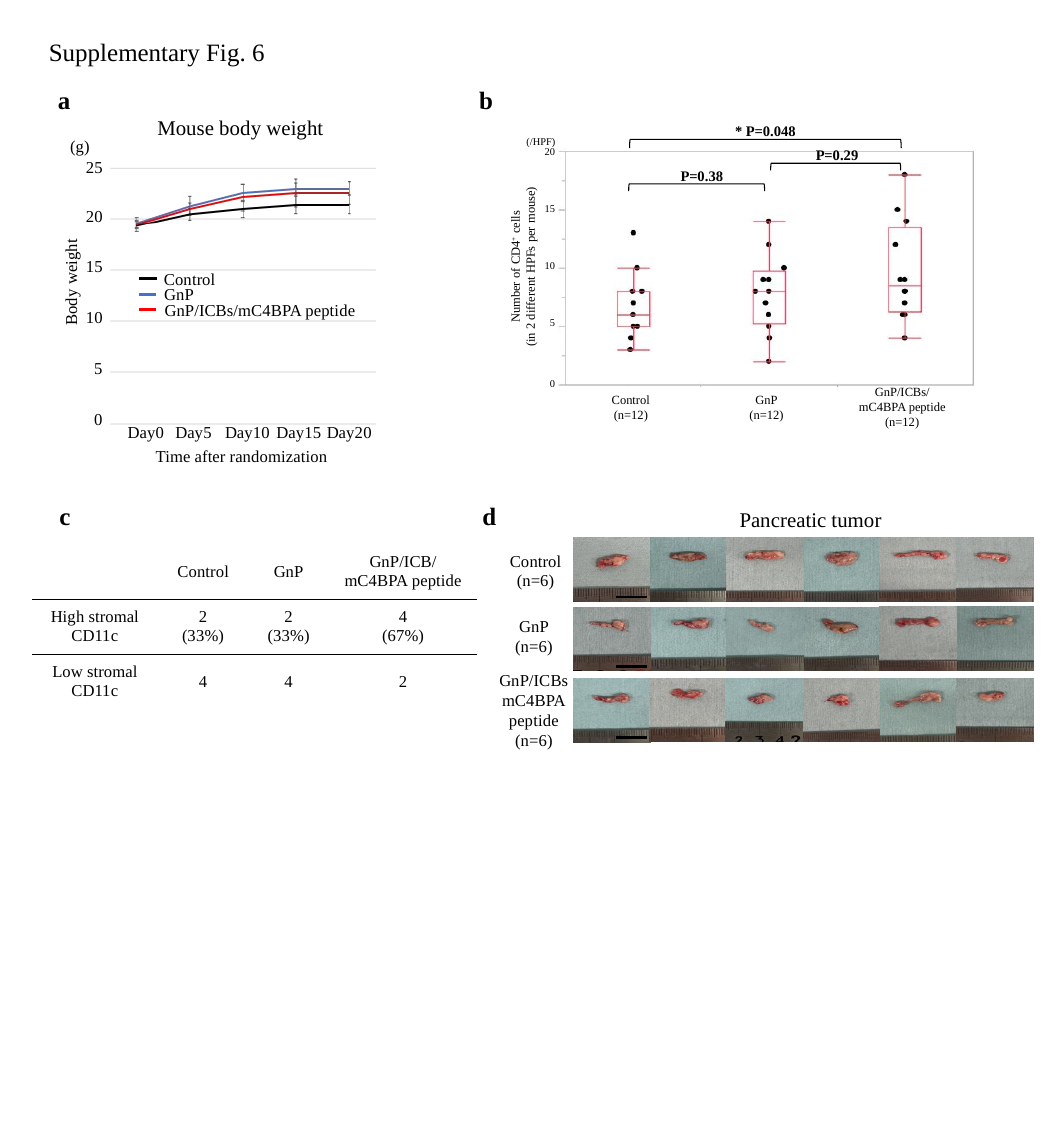

Supplementary Fig. 6
b
a
Mouse body weight
* P=0.048
(/HPF)
20
P=0.29
P=0.38
15
10
5
0
GnP/ICBs/
mC4BPA peptide
(n=12)
Control
(n=12)
GnP
(n=12)
Number of CD4+ cells
(in 2 different HPFs per mouse)
(g)
25
20
15
Control
Body weight
GnP
GnP/ICBs/mC4BPA peptide
10
5
0
Day5
Day15
Day0
Day10
Day20
Time after randomization
c
d
Pancreatic tumor
Control
(n=6)
| | Control | GnP | GnP/ICB/ mC4BPA peptide |
| --- | --- | --- | --- |
| High stromal CD11c | 2 (33%) | 2 (33%) | 4 (67%) |
| Low stromal CD11c | 4 | 4 | 2 |
GnP
(n=6)
GnP/ICBs
mC4BPA
peptide
(n=6)
