# Supplementary material for: C4b-binding protein α-chain enhances antitumor immunity by facilitating the accumulation of tumor-infiltrating lymphocytes in the tumor microenvironment in pancreatic cancer
Source: J Exp Clin Cancer Res. 2021 Jun 24;40:212. doi: 10.1186/s13046-021-02019-0 (PMC8228942; doi:10.1186/s13046-021-02019-0)
Supplement: Supplementary file 3 — Additional file 3. [file 13046_2021_2019_MOESM3_ESM.docx]

**Supplementary information**

**Supplementary Fig. 1.** Clinical outcomes of patients with PDAC based on immunohistochemical analysis of CD40 and CD8 expression in resected human PDAC tissues. (a) Patients in the high stromal CD40 group presented significantly longer overall survival (P=0.042, log-rank test) than did those in the low stromal CD40 group. (b) Kaplan-Meier analyses for overall survival of PDAC patients based on expression of CD8^+^ T cells. Patients in high CD8^+^ group presented significantly longer overall survival (P=0.0013, log-rank test) than those in the low CD8^+^ group. (c) IF for DAPI and alpha SMA (left); DAPI and C4BPA (middle); and DAPI, alpha SMA, and C4BPA (right) in human PDAC tissue.

**Supplementary Fig. 2.** Recombinant human C4BPA stimulation increases proliferation in CD40 expressing PDAC cells. (a) Western blot results showing C4BPA expression in the supernatant of human PDAC cell lines. (b) Confirmation of the knockdown of CD40 expression at 96 h (day 4), 120 h (day 5), and 144 h (day 6) after siRNA transfection by western blotting in PANC-1 and Capan-2 cells. (c) rhC4BPA stimulation increased cell proliferation in CFPAC-1, CD40 highly-expressing PDAC cells (*P < 0.05, Welch’s *t*-test). (d) Contrary, rhC4BPA stimulation did not alter cell proliferation in MIA PaCa-2, a CD40 low-expressing PDAC cell line. Results are represented as mean ± SD. Each experiment was performed at least three times. ns, not significant.

**Supplementary Fig. 3.** The cytotoxic efficacy in the combination of gemcitabine with C4BPA stimulation in human PDAC cells. In control groups, cell proliferation was significantly decreased by low-dose Gem exposure (at 20 and 50 ng/mL) in Capan-2 cells, but not in PANC-1 cells. Conversely, in rhC4BPA stimulation groups, cell proliferation was significantly decreased at 20 ng/mL Gem exposure in both PDAC cell lines and was not altered at 50 ng/mL compared with that in the control group.

**Supplementary Fig. 4.** mouse C4BPA expression and its peptide functions in mouse PDAC cells. (a) The mouse C4BPA (mC4BPA) peptide [54] which is composed of 54 amino acids from the C-terminus of mC4BPA (b) Western blot results showing mC4BPA and mouse CD40 (mCD40) expression in various mouse PDAC cell lines. (c) Representative scatter plots of mCD4^+^ and mCD8^+^ T cells in the control and mC4BPA peptide [54] stimulation groups analyzed using flow cytometry. (d) Number of mCD4^+^ and mCD8^+^ cells on each group. After mPBMCs were stimulated with mC4BPA peptide [54], the numbers of both mCD4^+^ and mCD8^+^ T cells were significantly increased compared with those in the control group (*P < 0.05, Welch’s *t*-test). (e) mC4BPA peptide stimulation increased cell proliferation in PKCY cells compared with that in control cells (*P < 0.05, Welch’s *t*-test). Results are represented as the mean ± SD. Each experiment was performed at least three times.

**Supplementary Fig. 5.** Comparisons of mouse body weight and CD11c expression in the stroma of mPDAC tumors between mC4BPA peptide group and control group. (a) The transition of body weights of the experimental mice during the study. (b) The staining patterns of CD11c^+^ cells (dendritic cells) in the stroma of mPDAC tumors were categorized into high (left) or low (right) stromal CD11c expression based on the intensity and area of expression. The boxed area is magnified in the lower panel. Original magnification: upper panels ×40, lower panels ×200. Bar, 50 µm. (c) The rate of high stromal CD11c expression in mC4BPA peptide group is higher than that in the control group in the stroma of mouse PDAC tumor.

**Supplementary Fig. 6.** Various parameters of the preclinical study. (a) The transition of body weights of the experimental mice during the study. (b) Number of CD4+ cells counting in 2 different high-power fields per mouse are depicted for the three groups in box-plot histograms (Control vs. GnP/ICBs/mC4BPA peptide: *P=0.048, Mann–Whitney–Wilcoxon test). (c) The rate of high stromal CD11c expression in the stroma of mPDAC tumors among these three groups. (d) All pancreatic tumors in the preclinical trial. Bar, 10 mm.
